# Pre‐operative radiological grading and inflammatory mediators as candidate biomarkers of early joint degeneration in patients with femoroacetabular impingement

**DOI:** 10.1002/jeo2.70923

**Published:** 2026-09-24

**Authors:** Eleonora Olivotto, Elisa Bubbico, Alessio Bucciarelli, Andrea Burla, Irene Tampieri, Giorgia Borciani, Stefano Zaffagnini, Enrico Tassinari

**Affiliations:** ^1^ RAMSES Laboratory, Research Centre Codivilla‐Putti, IRCCS Istituto Ortopedico Rizzoli Bologna Italy; ^2^ Orthopaedic‐Traumatology and Prosthetic Surgery and Revisions of Hip and Knee, IRCCS Istituto Ortopedico Rizzoli Bologna Italy; ^3^ BIOtech Research Center and European Institute of Excellence on Tissue Engineering and Regenerative Medicine, Department of Industrial Engineering University of Trento Trento Italy; ^4^ Department of Biomedical and Neuromotor Sciences—DIBINEM Alma Mater Studiorum University of Bologna Bologna Italy; ^5^ Orthopaedic and Traumatologic Clinic II, IRCCS Istituto Ortopedico Rizzoli Bologna Italy; ^6^ Present address: Department of Trauma and Orthopaedic Surgery Maggiore Hospital Largo Nigrisoli 2 40133 Bologna Italy

**Keywords:** biomarkers, femoroacetabular impingement, inflammatory molecules, osteoarthritis, synovial fluid

## Abstract

**Purpose:**

The aim of the study was to identify potential biomarkers of pre‐arthritic condition and early osteoarthritis (OA) in patients with femoroacetabular impingement (FAI), by analysing clinical data and quantifying inflammatory molecules, typically involved in OA pathology, in body fluids.

**Methods:**

FAI patients undergoing hip arthroscopy were enroled after providing written informed consent. Demographic data (age, sex, body mass index [BMI]) were collected, together with radiographic grading of hip degeneration (Tönnis scale) and intra‐articular pathology grading (Outerbridge classification). Cartilage degradation markers and immunomodulating molecules were quantified by commercially available sandwich‐enzyme‐linked assay and multiplex bead‐based sandwich immunoassay kits.

**Results:**

Forty‐nine patients were enroled, 38 synovial fluids (SFs) and urine samples were available. Median age 32 years ± 8.35 standard deviation (SD); median BMI 24 kg/m^2^ ± 4.89 SD. Higher BMI corresponded to acetabular chondropathy and was associated with increased C‐telopeptide fragments of Type II collagen (CTX‐II) release in both SFs and urine. Radiological and intra‐operative scores showed moderate‐to‐strong positive correlation with synovial CTX‐II and slight‐to‐strong positive correlation with vascular endothelial growth factor (VEGF). CTX‐II and Cartilage Oligomeric Matrix Protein (COMP) in SFs were positively correlated, showing a high‐to‐moderate positive correlation with VEGF. VEGF demonstrated a high‐to‐moderate positive correlation with interleukin (IL)‐6 and IL‐8. IL1‐β correlates moderately positively with IL‐8, MIP‐3β and TNF‐α, which in turn is linked to the release of 6kine, MIP‐3β and RANTES. Except for IL‐8, all chemokines showed a similar release pattern, suggesting a synergistic and continuous inflammatory mechanism.

**Conclusions:**

A higher BMI was associated with increased CTX‐II in both body fluids, suggesting a potential link between metabolic status and early cartilage degradation in FAI patients. Radiographic and intraoperative indicators of joint degeneration were also associated with selected synovial biomarkers (CTX‐II, VEGF, IL‐6, IL‐8). These findings suggest that synovial and urinary biomarkers may represent candidate markers of early joint degeneration in FAI, useful for clinical evaluation of FAI patients at risk of progression toward OA.

**Level of Evidence:**

Level IV, case series with no comparison group.

**Abbreviations**
BMI: body mass index
CCL: chemokine [C–C motif] ligand
CD: cluster of differentiation
CE‐AVEC: Comitato Etico Area Vasta Emilia Centro
COMP: Cartilage Oligomeric Matrix Protein
CTX‐II: C‐telopeptide fragments of Type II collagen
ELISA: enzyme‐linked assay
FAI: femoroacetabular impingement
HOA: hip osteoarthritis
IL: interleukin
MCP‐1: monocyte chemoattractant protein‐1
MIB‐3β: macrophage inflammatory protein‐3‐beta
OA: osteoarthritis
SD: standard deviation
SFs: synovial fluids
THR: total hip replacement
TNF: tumour necrosis factor
VEGF: vascular endothelial growth factor

## INTRODUCTION

Femoroacetabular impingement (FAI) is defined as a pathological mechanical process caused by morphologic alterations of the acetabulum or femur, leading to hip pain and limited range of motion [12, 24, 25, 32, 47]. The abnormal biomechanics between the femoral head and acetabulum cause impaired joint functionality, which in turn leads to mechanical impingement and degeneration of all joint tissue components, resulting in hip osteoarthritis (HOA) onset and progression. Two types of FAI are described: the pincer type, caused by abnormalities in acetabular shape or orientation and the cam type, resulting from morphological alterations of the proximal femur. A combined or mixed form occurs when both mechanisms are present [7, 12].

The treatment of the early stages of FAI and pre‐arthritic condition in adolescents and young adults is crucial to reduce the incidence of end‐stage OA and the need for total hip replacement (THR) [7]. Current management strategies vary from conservative treatments to surgical procedures, guided by patient‐specific variables including age, functional capacity, symptom burden and the condition of the affected tissues [13]. Hip arthroscopy has become an important surgical intervention for the treatment of many patients with early hip disease and has grown in popularity at an exponential rate over the past years [9, 37], being minimally invasive, associated with faster recovery and a lower incidence of complications [2, 17, 28, 33, 34, 35].

Of extreme importance, however, is also the identification of the inflammatory state of the joint in patients with FAI, detecting the early stages of hip inflammation and cartilage breakdown to recommend appropriate treatments before disease progression. Indeed, besides mechanical derangement, inflammation plays a key role in the pathogenesis and progression of OA [36, 42]. OA is not a prototypical degenerative disease resulting from normal bodily wear and tear, but rather a multifactorial disorder in which low‐grade, chronic inflammation plays a central role. This inflammation emerges early during OA, driven by interactions between the immune system and factors such as local tissue damage and metabolic dysfunction [26, 49]. Various soluble inflammatory mediators have been identified in OA joint tissues and fluids, including cytokines, chemokines, growth factors, adipokines, prostaglandins and leukotrienes [36]. Among the secreted inflammatory factors, interleukin [IL]‐1β, tumour necrosis factor [TNF]‐α and IL‐6 seem to be the main proinflammatory cytokines involved in the pathophysiology of OA [49]. In addition, certain chemokines (i.e., IL‐8, chemokine [C–C motif] ligand [CCL]‐5, CCL19) may contribute to the OA onset and progression [39] by enhancing the breakdown of cartilage matrix components and increasing the release of metabolic markers of cartilage degradation [27], including C‐telopeptide fragments of Type II collagen (CTX‐II) and cartilage oligomeric matrix protein (COMP) [15].

Also, growth factors such as the vascular endothelial growth factor (VEGF) have been implicated in OA, as they can promote angiogenesis and thereby facilitate the infiltration of immune cells into the joint, contributing to inflammation in OA [27].

In general, biomarkers are biological molecules present in body fluids or tissues that can be measured and evaluated as indicators of physiological and pathophysiological processes [3, 29]. Biomarkers of pre‐arthritic condition and early OA represent a major unmet need, and further research is required to identify biomarkers that characterize early events in OA pathogenesis.

Therefore, the assessment of biomarkers associated with FAI that are also present in OA [27], may show a clinical utility in the identification of progression towards hip OA, as these joint alterations often precede the radiographic evidence of the disease [16].

The purpose of this exploratory study was to evaluate associations between selected synovial and urinary biomarkers of cartilage degradation and inflammation, typically involved in OA pathology, and demographic, radiographic and intraoperative indicators of early joint degeneration in patients undergoing arthroscopy for FAI.

## METHODS

### Patient recruitment, demographic and clinical data collection

Consecutive patients with FAI undergoing hip arthroscopy were enroled within the framework of a comparative and randomized study conducted by the authors [43], where the use of cortisone intra‐articular injection at the end of arthroscopy was compared to hydrolyzed collagen peptides to investigate potential associations among preoperative symptoms and hip function, outcomes after arthroscopic surgery and the presence of inflammatory biomarkers in SFs. The present study is a case series with no comparison group, as all clinical and laboratory data were collected before surgery, with any influence of randomization. All participants provided written informed consent prior to inclusion. This study was approved and conducted in accordance with the guidelines of the Local Ethical committee (Prot. gen. 0008219) and conducted in accordance with the Declaration of Helsinki. Inclusion and exclusion criteria are reported in our previously published study [43]. Briefly, inclusion criteria were age 18–65 years and clinical diagnosis of symptomatic FAI. Exclusion criteria were listed as follows: inability to provide informed consent; dysplastic hips (lateral centre‐edge angle: <20°), malignancies or overall poor general condition of health; history of tumour or infection; established diagnosis of rheumatic diseases; arthroscopic surgery performed for any other reason than FAI and/or labral pathology; previous operations at the affected hip or clinical and radiographic signs of HOA (Tönnis classification >2); history of diabetes and neurologic disease; pregnant or breastfeeding women.

Demographic data (age, sex and body mass index [BMI]) were collected for all participants. Plain anteroposterior radiographs in standing position were obtained before surgery to evaluate the progressive degrees of degenerative changes to the hip using the Tönnis scale [44]. Macroscopic intra‐articular pathology was graded using the Outerbridge score for chondral lesions [30].

### Sample collection

SF was collected from patients by aspiration immediately before arthroscopy, centrifuged, aliquoted and stored at −80°C until analysis. Urine samples were likewise collected and stored at −80°C for long‑term preservation. Prior to analysis, urine samples were thawed gradually at room temperature and centrifuged.

### Biomarkers analysis

Inflammatory biomarkers involved in OA pathology, such as cartilage degradation markers and immunomodulating molecules, were analysed.

C‐telopeptide fragments of Type II collagen (CTX‐II) and Cartilage Oligomeric Matrix Protein (COMP), two sensitive markers of cartilage degradation, were quantified in SFs and urine using commercially available sandwich‐ enzyme‐linked assay (ELISA) Kits (Elabscience Biotechnology Inc.).

Cytokines (IL‐6, IL‐1β, TNF‐α) and chemokines (IL‐8, CCL2/monocyte chemoattractant protein‐1 [MCP‐1], CCL5/RANTES, CCL19/macrophage inflammatory protein‐3‐beta [MIP‐3β] and CCL21/6kine) were simultaneously quantified in the SFs at basal condition with commercially available multiplex bead‐based sandwich immunoassay kits (Bio‐Rad Laboratories). Data were analysed using the Bio‐Plex Manager software version 6.0 (Bio‐Rad Laboratories).

The mediator of both angiogenesis and vasculogenesis, VEGF, was also quantified in SFs with a sandwich‐ELISA Kit (Human VEGF Quantikine ELISA Kit; R&D Systems, Inc.).

The kit standard curves were fit using a four‐parameter logistic model, and unknown concentrations were calculated from these curves based on the optical densities measured with an Infinite® M Plex microplate reader (Tecan Trading AG). The equations used for curve fitting have been previously described [43].

### Statistical analysis

**Figure 1:**
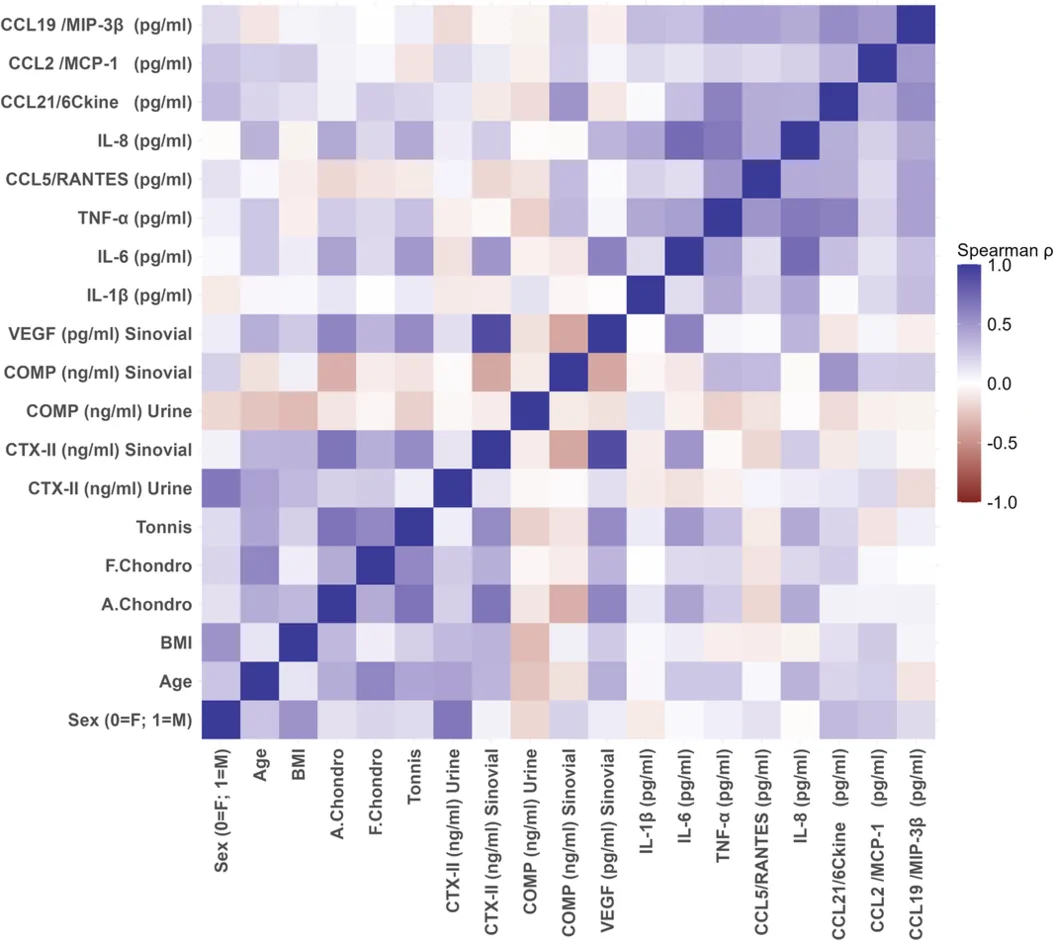
Spearman correlation matrix of clinical data and inflammatory molecules (pg/mL) detected in synovial fluids and urine of FAI patients (diverging scale centred at 0 with a range −1 to 1). BMI, body mass index; CCL, chemokine [C–C motif] ligand; CTX‐II, C‐telopeptide fragments of Type II collagen; COMP, Cartilage Oligomeric Matrix Protein; F, female; FAI, femoroacetabular impingement; IL, interleukin; M, male; TNF, tumor necrosis factor; VEGF, vascular endothelial growth factor.

Analyses were performed in R (v. 4.5.1) using the packages readxl, Hmisc, ggplot2, reshape2, rlang and the RStudio IDE (v.2023.06.1). Only numeric variables were retained; variables with fewer than three non‐missing observations or with zero variance were excluded to avoid unstable estimates. Pairwise analyses used all available cases after omitting missing values for the two variables involved (pairwise complete observations). Because several variables were ordinal or non‐normally distributed, associations were quantified with Spearman's rank correlation (*ρ*). Both the correlation matrix and the corresponding matrix of asymptotic *p* values were computed (Figure 1 and Supporting Information S1: Figure 1). Given the exploratory, hypothesis‐generating nature of the study, no adjustment for multiple comparisons was applied; *p* values are therefore reported as unadjusted and descriptive rather than confirmatory. Associations were interpreted primarily on the basis of effect size (*ρ*) and of their consistency across related variables and across the two biological fluids, rather than on statistical significance alone. Two heatmaps were generated with: a correlation heatmap with a diverging scale centred at 0 (range −1 to 1), and a *p* value heatmap spanning 0 to 1. For visualization of individual relationships, scatterplots were produced for every variable pair with *p* < 0.10.

## RESULTS

### Patient demographics and baseline characteristics

Among the 49 consecutive patients undergoing arthroscopy for FAI who were enroled in the study, SF samples were available for the analysis in 38 cases. For consistency, only urine samples from these same patients were included in the comparative analysis. Moreover, 20 patients had an initial diagnosis of cam‐type, four had pincer‐type, and the remaining patients had a mixed combination of the two morphologies. Patient demographics and clinical features at baseline are summarized in Table 1.

**Table 1:** Descriptive statistics of the patient population (38 patients).

Variable	Unit of measurement	Value
Male/Female	Number (%)	21 (55)/17 (45)
Age (mean ± SD)	Years	32 ± 8.35
BMI (mean ± SD)	kg/m^2^	24 ± 4.89
TöNNIS scale (0–3)	Scale value, number of patients (%)	0, 23 (60)
		1, 13 (34)
		2, 2 (6)
		3, 0 (0)
Acetabular chondropathy	Number of patients (%)	13 (34)
Femoral chondropathy	Number of patients (%)	5 (13)
Acetabular and femoral chondropathy yes/no	Number of patients (%)	19 (50)/1 (3)

Abbreviations: BMI, body mass index; SD, standard deviation.

### Biomarkers analysis and correlation with demographic and clinical data

A Spearman correlation matrix was performed as an exploratory data analysis with both biological and demographic/clinical data to evaluate the presence of correlations (Figure 1).

Related to the demographic data, the sex of patients had a slight positive correlation with age (*ρ* = 0.3), as females are younger compared to males (mean and standard deviation [SD] 30 ± 9.51 years vs. 34 ± 6.95, respectively). As expected, sex had a moderate, highly statistically significant positive correlation with BMI (*ρ* = 0.5, *p* = 0.0008), which was higher in male patients (mean and SD 26.6 ± 5 kg/m^2^ vs. 21.7 ± 3.16, respectively). A slight positive correlation was observed between sex and two chemokines (*ρ *= 0.3), which reached statistical significance for 6kine (*p* = 0.045) but not for MCP‐1, showing higher levels in males. A high positive correlation was also observed between sex and urinary CTX‐II levels, even if not statistically significant.

BMI was positively correlated with acetabular chondropathy (*ρ* = 0.3, *p* = 0.041). Between the markers of cartilage degradation, CTX‐II positively correlates with BMI in both SF and urine samples (*ρ* = 0.4, *p* = 0.027 and *ρ* = 0.3, *p* = 0.042, respectively). In contrast, urinary COMP showed a weak inverse correlation with BMI, but inversely (*ρ* = −0.3), not statistically significant. As for the synovial inflammatory biomarkers, there was a low positive correlation with VEGF and MCP‐1 (*ρ* = 0.3).

Correlation between intra‐operative macroscopic and radiological scores highlighted a moderate, statistically significant positive correlation between the acetabular and the femoral chondropathy (*ρ* = 0.4, *p* = 0.014). While acetabular chondropathy had a high but not significant correlation with the Tönnis score (*ρ* = 0.7), the femoral chondropathy showed a moderate but highly significant correlation with the score (*ρ* = 0.6, *p* = 0.00014).

Both the intra‐operative macroscopic and radiological scores showed a positive correlation with the synovial CTX‐II marker: The femoral chondropathy showed a moderate, highly statistically significant correlation (*ρ* = 0.4, *p* = 0.019); the acetabular chondropathy and the Tönnis score showed a strong, highly statistically significant (*ρ* = 0.7 and 0.6, respectively, with *p* < 0.0001). Only acetabular chondropathy showed a negative, non‐significant correlation with the other synovial degeneration marker COMP.

Among the synovial inflammatory biomarkers, both intra‐operative macroscopic and radiological scores showed positive correlations with VEGF: acetabular chondropathy and the Tönnis score (*ρ* = 0.6 with *p* < 0.0001 and 0.0002, respectively) demonstrated strong and highly statistically significant associations, while the femoral chondropathy showed a slight but significant correlation (*ρ* = 0.3 with *p* < 0.031). IL‐6 and IL‐8 showed a moderate positive correlation with acetabular chondropathy (*ρ* = 0.4 with *p* = 0.006 and 0.01, respectively) and with the Tönnis score (*ρ* = 0.5 and 0.4 with *p* < 0.0016 and 0.01, respectively).

### Correlation among inflammatory biomarkers

Regarding the simultaneous presence of cartilage degradation markers in the urine and inflammatory biomarkers in the SF samples, neither CTX‐II nor COMP showed any correlation.

On the contrary, synovial CTX‐II showed a moderate, statistically significant correlation with synovial COMP and IL‐6 (*ρ* = 0.4 and 0.5 with *p* = 0.01 and 0.001, respectively). A very high positive correlation between synovial CTX‐II and VEGF was observed, but not statistically significant (*ρ *=0.9). Synovial COMP showed moderate, statistically significant positive correlations with VEGF and 6Ckine (*ρ *=0.4 and 0.5, *p* = 0.0014 and 0.0009, respectively) and weak but significant correlations with RANTES and TNF‐α (*ρ *=0.3, *p* = 0.048 and 0.038, respectively). VEGF had a high and moderate statistically significant positive correlation with IL‐6 (*ρ *=0.6, *p* < 0.0001) and IL‐8 (*ρ *=0.4, *p* = 0.029), respectively.

IL‐1β showed a synergic release statistically significant with IL‐8, TNF‐α and MIP‐3β (*ρ *=0.4, 0.4 and 0.3; *p* = 0.008, 0.009 and 0.05, respectively). Both IL‐6 and IL‐8 had a positive correlation with the same three inflammatory molecules: TNF‐α moderate and high (*ρ *=0.5 and 0.6; *p* = 0.003, and *p* < 0.0001, respectively); 6Ckine low and moderate (*ρ *=0.3 and 0.4; *p* = ns, and *p* = 0.017, respectively); MIP‐3b low and moderate (*ρ *=0.3 and 0.4; *p* = ns, and *p* = 0.013, respectively). Otherwise, IL‐6 had a high positive correlation with IL‐8, but not statistically significant (*ρ *=0.7), while IL‐8 demonstrated a moderately significant correlation with RANTES (*ρ *=0.4, *p* = 0.014).

RANTES showed a moderate and statistically significant correlation with TNF‐α and the two chemokines 6Ckine and MIP‐3β (*ρ *=0.5, 0.4 and 0.5; *p* = 0.001, 0.015 and 0.004, respectively).

TNF‐α also showed a statistically significant positive correlation with those two chemokines: high for 6Ckine (*ρ *=0.6, *p* < 0.0001) and moderate for MIP‐3β (*ρ *=0.5, *p* = 0.004).

6Ckine had a moderate statistically significant positive correlation with MCP‐1 (*ρ *=0.4, *p* = 0.030) and a high with MIP‐3β (*ρ *=0.6, *p* = 0.0003) which in turn moderately correlated with MCP‐1 (*ρ *=0.5, *p* = 0.002).

## DISCUSSION

Reliable biomarkers of joint degeneration typically involved in OA pathology may improve risk stratification in patients with FAI, particularly in the pre‐arthritic phase. In this exploratory study, selected synovial and urinary markers of cartilage degradation and inflammation were associated with demographic, radiographic and intraoperative indicators of early joint degeneration in patients undergoing arthroscopy for FAI [4, 27, 45].

The main finding was that higher BMI and greater radiographic or intraoperative degenerative changes were associated with increased levels of CTX‐II and selected inflammatory mediators, supporting their potential role as candidate biomarkers in this patient population.

Demographic analysis revealed no significant difference between sexes: The median age was similar and as predictable; the BMI was higher in male patients. Regarding sex‐related differences in the SFs composition, males showed higher levels of 6kine, MCP‑1 and urinary CTX‑II. This is in line with the findings of Kriegova et al., who reported different levels of inflammatory cytokines as well as pro‐inflammatory mediators between male and female patients with knee OA [23]. Sex differences may arise from multiple factors (i.e., biomechanical properties, gene expression, behaviour, sex hormones) [10]; therefore, further preclinical studies are needed to improve the understanding of sex differences.

Higher BMI was associated with acetabular chondropathy in this cohort. Although this finding may be influenced by the predominance of cam and mixed‐type FAI, morphology‐specific analyses were not performed, and a causal relationship cannot be established. BMI was also positively associated with CTX‐II levels in both synovial fluid (SF) and urine. Since CTX‐II is considered a marker of cartilage degradation in OA, this association may suggest a link between metabolic status and early cartilage matrix turnover in patients with FAI [1].

Moreover, BMI was also positively associated with CTX‐II levels in both SF and urine, which is a well‐known marker of cartilage degradation in OA [15]. Conversely, BMI did not correlate significantly with COMP, a marker more closely related to OA severity/progression [11].

Interestingly, despite only 37% of patients being overweight (median BMI = 28), higher BMI was associated with increased release of pro‐inflammatory molecules such as VEGF and MCP‐1. These findings are consistent with previous evidence suggesting that overweight and obesity may be associated with a low‐grade inflammatory state [21]. However, the present data should be interpreted as exploratory and do not demonstrate that BMI directly induces cartilage breakdown or synovial inflammation. Overall, these results suggest that BMI may be a clinically relevant factor to consider when evaluating early degenerative changes in FAI patients. In our previous study, the synovial tissue analyses in FAI patients frequently revealed signs typical of the low‐grade synovitis found in early OA cases. We also found that the presence of mononuclear cell infiltration, typical of end‐stage OA, was associated with the worst clinical outcomes, suggesting that it might contribute to worsening the clinical condition after hip arthroscopy [45].

As already described, higher BMI in FAI patients is associated with a greater presence of cartilage wear [31] and increased susceptibility to hip OA [19]. For all these aspects described above, a high BMI is a risk factor that must be monitored in FAI patients, who may be considered in a ‘pre‐arthritic’ state.

A positive correlation was observed between intra‐operative cartilage grading, at the acetabular and femoral side and pre‐operative radiographic grading of cartilage loss using the Tönnis grading schemes, confirming findings by Jardon et al. [18]. Notably, cartilage loss is the strongest predictor of hip arthroscopy failure, as patients with high‐grade chondral loss demonstrate poorer outcomes [8].

To confirm the previous observation, both the intra‐operative macroscopic and radiological scores showed a positive correlation with fragments from matrix degradation, which could activate chondrocytes and synovial fibroblasts, leading to the induction of pro‐inflammatory molecules [5, 48]. Both scores showed a moderate‐to‐strong positive correlation with the synovial CTX‐II marker.

Interestingly, VEGF also correlates positively with both scores, in a strong and highly statistically significant manner for the acetabular chondropathy and the Tönnis score, slightly but significantly for femoral chondropathy. VEGF is involved in OA‐specific pathologies, including cartilage degeneration, osteophyte formation, subchondral bone cysts and sclerosis, synovitis and pain [14].

Acetabular chondropathy and Tönnis score were also moderately associated with the other two key inflammatory molecules implicated in OA pathophysiology [22, 24] as IL‐6 and IL‐8.

Therefore, given the importance of pre‐surgical assessment of cartilage damage for appropriate patient selection and good patient outcomes, the pre‐operative radiological grade may be used as a ‘first level’ biomarker of early signs of OA in FAI patients.

OA is a complex disease characterized by pathological changes across all the joint tissues and is increasingly described using several overlapping phenotypes, including the post‐traumatic phenotype [6]. FAI has a prominent aetiological role in the development of early HOA [12], leading to the development of early cartilage and labral damage, which results in the release of mediators with the activation of different inflammatory pathways [38, 46]. The evaluation of the inflammatory environment in FAI patients, and the identification of a possible synergy among the inflammatory molecules, is of extreme importance to prevent the onset and progression towards the end‐stage disease.

The release of the two cartilage degradation markers, CTX‐II and COMP, in the SFs was positively correlated, and both showed a moderate‑to‑strong positive correlation with VEGF, demonstrating their pro‐inflammatory action. The increase in CTX‐II was also positively correlated with IL‐6, one of the main proinflammatory cytokines involved in OA pathophysiology alongside IL‐1β and TNF‐α.

COMP had the same increase in release as TNF‐α and other chemokines belonging to the ‘C–C’ chemokine family (MIP‐3β and RANTES), which influence the recruitment of monocytes, lymphocytes and eosinophils [40]. Together, CTX‐II and COMP, through the interaction with different molecules, appear to contribute to a vicious cycle of inflammation and damage of hip joint tissues.

Interestingly, VEGF correlated strongly with IL‑6 and moderately with IL‑8, a mediator of synovial inflammation, and therefore it sustains the ‘low‐grade’ synovitis [41]. IL‐6 and IL‐8 showed a high correlation between themselves and a moderate correlation with the other chemokines, that is, 6kine and MIP‐3β, low with MCP‐1 and moderate‐to‐high, respectively, with TNF‐α.

IL1‐β and TNF‐α are the major players among the proinflammatory cytokines involved in OA [20], acting independently or synergistically with other cytokines. Indeed, IL1‐β correlates moderately positively with IL‐8, MIP‐3β and TNF‐α, which in turn are linked to the release of 6kine‐MIP‐3β and RANTES. Except for IL‐8, all the chemokines showed similar release patterns, suggesting a synergistic and continuous mechanism that perpetuates inflammation.

Although the exploratory nature of the study, the relatively small sample number of SF samples included in the final analysis may represent a limitation in terms of reproducibility of some associations. Therefore, future studies with larger cohorts will be necessary to confirm and strengthen the observed data. Another potential limitation may be related to the SF biomarker detection: Multiplex bead‐based flow cytometry enables simultaneous quantification of multiple markers in the same sample, but the wide variability in biomarker concentrations across biological fluids may affect the detection of all the targets within the same sample in any case.

Our study confirms the simultaneous release of multiple inflammatory molecules in the SFs of FAI patients, highlighting that the evaluation of the inflammatory environment in FAI is of extreme importance to understand the early joint degeneration and prevent progression towards the end‐stage disease. The potential clinical implications of the findings presented in our study might contribute to risk stratification for progression toward OA. Therefore, the molecules analysed in the SFs may be used as ‘second‐level’ biomarkers to monitor biological responses and the post‐operative outcomes after arthroscopy.

A further limitation is that a large number of pairwise correlations were tested without correction for multiple comparisons, which increases the risk of Type I error; some of the reported associations may therefore represent chance findings. A formal multiplicity correction (e.g., Bonferroni or false‐discovery‐rate methods) was deliberately not applied, since the aim was hypothesis generation rather than confirmatory testing, and such an adjustment would have substantially inflated the Type II error rate in a cohort of this size. Consequently, all the associations reported here should be regarded as preliminary and require confirmation in larger, adequately powered, prospective cohorts with pre‐specified hypotheses.

## CONCLUSION

This exploratory study found that higher BMI was associated with increased CTX‐II levels in both SF and urine, suggesting a potential link between metabolic status and early cartilage matrix degradation in patients with FAI. Radiographic and intraoperative indicators of joint degeneration were also associated with selected synovial biomarkers, particularly CTX‐II, VEGF, IL‐6 and IL‐8.

These findings support the hypothesis that synovial and urinary biomarkers may represent candidate markers of early joint degeneration in FAI.

However, given the cross‐sectional nature of the study and the relatively small number of SF samples analysed, any potential clinical application must be interpreted with caution.

Longitudinal prospective studies with larger cohorts are required to validate the diagnostic and prognostic value of these biomarkers and to determine whether they can reliably monitor postoperative outcomes after hip arthroscopy.

## AUTHOR CONTRIBUTIONS

**Eleonora Olivotto**: Substantial contributions to the conception and design of the work; preparation of documents for IRB approval; acquisition, analysis and interpretation of data; sample collection; synovial fluid analysis; drafting the work and revising it critically for important intellectual content; final approval of the version to be published; agreement to be accountable for all aspects of the work in ensuring that questions related to the accuracy and integrity of any part of the work are appropriately investigated and resolved. **Elisa Bubbico** and **Irene Tampieri**: Sample and data collection; final approval of the version to be published. **Alessio Bucciarelli**: Statistical analysis and interpretation of data; drafting the work and revising it critically for important intellectual content; final approval of the version to be published. **Andrea Burla**: Sample collection; drafting the work and final approval of the version to be published. **Giorgia Borciani**: Drafting the work and revising it critically for important intellectual content; final approval of the version to be published. **Stefano Zaffagnini**: Drafting the work and revising it critically for important intellectual content; final approval of the version to be published; agreement to be accountable for all aspects of the work in ensuring that questions related to the accuracy and integrity of any part of the work are appropriately investigated and resolved. **Enrico Tassinari**: Substantial contributions to the conception and design of the work; acquisition, analysis and interpretation of data; patients' selection and performing arthroscopy procedure and sample collection; drafting the work and revising it critically for important intellectual content; final approval of the version to be published; agreement to be accountable for all aspects of the work in ensuring that questions related to the accuracy and integrity of any part of the work are appropriately investigated and resolved.

## FUNDING INFORMATION

The authors have no funding to report.

## CONFLICT OF INTEREST STATEMENT

The authors declare no conflicts of interest.

## ETHICS STATEMENT

The study was approved by the ‘Comitato Etico di Area Vasta Emilia Centro della Regione Emilia‐Romagna (CE‐AVEC)’ Prot. gen. 0008219. All patients enroled provided written informed consent.

## Supporting information

Supporting File 1.

## Data Availability

The data that support the findings of this study are available from the first author, Eleonora Olivotto, upon reasonable request.
