# Supplementary material for: Pre‐operative radiological grading and inflammatory mediators as candidate biomarkers of early joint degeneration in patients with femoroacetabular impingement
Source: J Exp Orthop. 2026 Sep 24;13(3):e70923. doi: 10.1002/jeo2.70923 (PMC13604036; doi:10.1002/jeo2.70923)
Supplement: Supplementary file 1 [file JEO2-13-e70923-s001.docx]

**Title: Pre-operative radiological grading and inflammatory mediators as candidate biomarkers of early joint degeneration in patients with femoroacetabular impingement**

**Authors:**

Eleonora Olivotto^1^, Elisa Bubbico^2^, Alessio Bucciarelli^3^, Andrea Burla^4^, Irene Tampieri^4^, Giorgia Borciani^1^, Stefano Zaffagnini^5^, Enrico Tassinari^5^

**Affiliations:**

^1^RAMSES Laboratory, Research Centre Codivilla-Putti, IRCCS Istituto Ortopedico Rizzoli, Bologna (Italy)

^2^Orthopaedic-Traumatology and Prosthetic Surgery and Revisions of Hip and Knee, IRCCS Istituto Ortopedico Rizzoli, Bologna (Italy)

^3^BIOtech Research Center and European Institute of Excellence on Tissue Engineering and Regenerative Medicine, Department of Industrial Engineering, University of Trento, Trento (Italy)

^4^Department of Biomedical and Neuromotor Sciences DIBINEM, Alma Mater Studiorum University of Bologna, Bologna, Italy

^5^Orthopaedic and Traumatologic Clinic II, IRCCS Istituto Ortopedico Rizzoli, Bologna (Italy)

**Corresponding Author:**

Name: Andrea Burla

Email: andrea.burla@studio.unibo.it

**Supplementary Figure 1. Matrix of asymptotic p-values of clinical data and inflammatory molecules (pg/ml or ng/ml) detected in synovial fluids and urine of FAI patients (diverging scale cantered at 0 with a range −1 to 1) (F. condro = femural condropathy; A.condro = acetabular condropathy; BMI = body max index; F = female; M = male).**
